# Brivaracetam for spinal cord injury–related neuropathic pain: results of a pilot double-blinded, randomized, placebo-controlled clinical trial

**DOI:** 10.1097/PR9.0000000000001301

**Published:** 2025-06-16

**Authors:** Leslie R. Morse, Ricardo A. Battaglino, Nguyen Nguyen, Brian DeVries, Abigail Welch, Ana Lucia Battaglino, Clas Linnman, Michael Stillman, Robert Wudlick, Joda Glossner, Grant Anderton, Richard Goldstein, Scott P. Falci

**Affiliations:** a Christine E. Lynn Rehabilitation Center, Department of Physical Medicine and Rehabilitation, University of Miami Miller School of Medicine, Miami, FL, USA; b Department of Rehabilitation Medicine, University of Minnesota Medical School, Minneapolis, MN, USA; c Craig Rehabilitation Hospital, Englewood, CO, USA; d Spaulding Neuroimaging Laboratory, Spaulding Rehabilitation Hospital, Harvard Medical School, Charlestown, MA, USA; e Sidney Kimmel Medical College, Thomas Jefferson University, Philadelphia, PA, USA; f RxArtisans Compounding Pharmacy, Wayzata, MN, USA; g Swedish Hospital, Englewood, CO, USA

**Keywords:** Spinal cord injury, Rehabilitation, Neuropathic pain, Therapeutics, Clinical trial

## Abstract

**Objective:**

Assess feasibility, safety, and tolerability of 3 months of oral brivaracetam for severe (9–10/10) spinal cord injury–related neuropathic pain.

**Methods:**

We conducted a multisite pilot study where adults were randomized to 3 months of brivaracetam (100 mg BID) or placebo. Pain was assessed via the Brief Pain Inventory, the International Spinal Cord Injury Pain Basic Dataset (version 2.0), and a daily pain diary.

**Results:**

We enrolled 24 participants across 2 sites and 14 participants were included in the final analysis (n = 8 placebo, n = 6 brivaracetam). The drug was tolerated with expected, nonserious side effects (nausea if taken on an empty stomach, fatigue that improved after 2–3 days). Side effects were more common in the brivaracetam group (73%, n = 8 vs 33%, n = 3). We observed a mean 2.7-point reduction in daily worst pain in the brivaracetam group compared to a 1.0-point reduction in the placebo group. The percentage of participants experiencing 30% (100% vs 60%) and 50% (60% vs 12.5%) pain reduction was greater in the brivaracetam group.

**Conclusions:**

These findings suggest safety, tolerability, and feasibility of brivaracetam for treating neuropathic pain in spinal cord injury. These pilot findings will be used to power future efficacy clinical trials.

## Abstract 2

Supplemental Digital Content is Available in the Text.

This pilot study suggests the feasibility of conducting a fully powered clinical trial of brivaracetam for treating neuropathic pain in spinal cord injury.

## 1. Introduction

The global prevalence of spinal cord injury (SCI) is more than 20 million people.^20^ Roughly 60% experience long-term neuropathic pain that interferes with rehabilitation and quality of life.^2^ According to current international guidelines, first-line agents for neuropathic pain in adults include tricyclic antidepressants and gabapentinoids,^8^ but these are often ineffective for SCI-neuropathic pain. Despite weak clinical trial data supporting strong opioid use for neuropathic pain,^8^ roughly 40% of individuals with SCI-neuropathic pain reported chronic opioid use.^24^ Cannabinoids are also routinely used for neuropathic pain relief after SCI despite a weak recommendation for use as a “D treatment option” in the revised CanPain SCI clinical practice guidelines.^3,14,18^ The experience of living with severe neuropathic pain has been described as, “…*chronic, debilitating, and depressing; at times leaving me hopeless and nearing the end of my will to live*” (SCI Lived Experience Consultant).

Targeting mechanisms of neuropathic pain may identify effective therapies. The synaptic vesicle glycoprotein 2A (SV2A) is a ubiquitous synaptic vesicle protein that is the target of anticonvulsant drugs that are used to tread neuropathic pain, including levetiracetam.^11^ Brivaracetam, which binds SV2A, is an FDA-approved drug to treat partial-onset seizures.^13^ Because brivaracetam has 30 times the SV2A-binding affinity compared to levetiracetam,^13^ we hypothesized that brivaracetam would be effective at reducing severe neuropathic pain. We, therefore, conducted a randomized, placebo-controlled, multisite pilot clinical trial to determine tolerability and safety in adults with SCI. We compared various pain tools to determine which measure would best reflect responsiveness to brivaracetam treatment for use in future trials.

## 2. Materials and methods

### 2.1. Participants and clinical trial design

**Figure 1.**
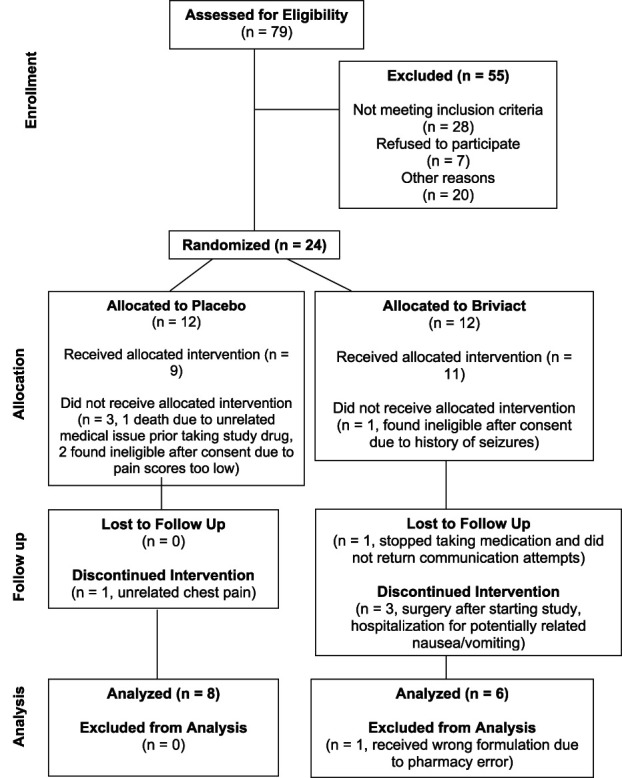
Study flow (CONSORT diagram).

We conducted a multicenter pilot clinical trial assessing feasibility and tolerability of brivaracetam for severe neuropathic pain, rated 9 to 10 on a 0 to 10 Numerical Rating Scale (NRS). Enrollment and testing occurred from September 20, 2021 (first participant enrolled) to May 8, 2023 (all study assessments completed) at 2 neurorehabilitation centers in the United States. Adults with SCI were randomized via simple randomization using a random number table to 3 months of brivaracetam (100 mg BID) or placebo. Participants and all study personnel were blinded. Treatment assignment was known only to the investigational drug pharmacy. This trial was registered at clinicaltrials.gov (NCT04379011) and completed under IND# 150388. The protocol was approved by our Institutional Review Board (IRB), and all participants gave written informed consent. An external medical monitor reviewed all serious adverse events to determine study relatedness. We recruited participants nationally via implementation of e-consenting, virtual visits, and overnight delivery of study drug. Eligibility criteria are presented in Table 1. We enrolled 24 participants across 2 sites (see Fig. 1, 12 at site A and 12 at site B). Three participants were found to be ineligible after screening and were removed from the study (pain too low: IDs 3008 and 3013, and active seizures: ID 4031). One participant (ID 4017, placebo) died of unrelated causes after enrolling and before starting the study. Twenty enrolled participants received at least a partial course of medication (n = 9 placebo and n = 11 brivaracetam). Demographic and Adverse Event information is presented for these 20 participants (Tables 2 and 3).

**Table 1:** Study criteria.

Inclusion criteria
18 y of age or older
Completed inpatient rehabilitation and living in the community
Ongoing severe below-level neuropathic pain (daily average 9/10 or 10/10)
Tried and failed to achieve adequate pain relief with the use of other drugs (previous pain management drugs failed to decrease their pain below a self-reported level of 9 out of 10)
For women of child-bearing potential: currently practicing an effective form of 2 types of birth control (defined as those, alone or in combination, that result in a low failure rate [ie, less than 1% per year] when used consistently and correctly)
Exclusion criteria
Progressive myelopathy secondary to posttraumatic cord tethering or syringomyelia
Active use of drugs known to interact with brivaracetam: rifampin, carbamazepine, sodium oxybate, buprenorphine, propoxyphene, levetiracetam, and phenytoin
Contraindications to brivaracetam or pyrrolidine derivatives including allergy
History of malabsorption or other gastrointestinal (GI) disease that may significantly alter the absorption of brivaracetam
Brain injury or cognitive impairment limiting the ability to follow directions or provide informed consent
Pregnancy or lactation
Epilepsy or active treatment for seizure disorder
Past or current active suicidality
Untreated psychiatric disease
Drug addiction
Moderate or heavy alcohol intake (up to 4 alcoholic drinks for men and 3 for women in any single day, and a maximum of 14 drinks for men and 7 drinks for women per week)
Hepatic cirrhosis, Child-Pugh grades A, B, and C
Impaired renal function
Active, clinically significant disease (eg, renal, hepatic, neurological, cardiovascular, pulmonary, endocrine, psychiatric, hematologic, urologic, or other acute or chronic illness) that, in the opinion of the investigator, would make the patient an unsuitable candidate for this trial
Use of any investigational drug 30 d before enrollment in this study
MRI exclusion Criteria*
Retained bullet fragments
Noncompatible metal implants
Implanted devices such as non-MRI compatible baclofen pumps

* People who met the MRI Exclusion Criteria were still eligible for the study but did not participate in MRI scanning.

**Table 2:** Demographics of enrolled participants.

	Placebo (n = 9)	Brivaracetam (n = 11)	*P*
Demographics			
Age (y) [mean ± SD]	51.6 ± 7.9	43.5 ± 12.5	0.11
Years postinjury (y) [mean ± SD]	10.2 ± 8.8	17.1 ± 9.8	0.12
Males, n (%)	8 (88.9%)	6 (54.6%)	0.16
White, n (%)	8 (88.9%)	10 (90.9%)	1.0
Wheelchair users, n (%)	5 (55.6%)	9 (81.8%)	0.34
Injury completeness, n (%)			
Motor complete paraplegia (AIS A/B)	3 (33.0%)	5 (45.0%)	0.67
Baseline pain measures			
Worst pain in past 24 h [mean ± SD]	8.7 ± 1.1	9.6 ± 0.7	0.03
Least pain in past 24 h [mean ± SD]	5.0 ± 2.7	5.6 ± 2.5	0.60
Average pain in past 24 h [mean ± SD]	6.7 ± 1.7	7.7 ± 1.3	0.14
Pain right now [mean ± SD]	5.6 ± 2.2	7.8 ± 2.0	0.03
Douleur Neuropathique en 4 questions (DN4) score [n]	6.4 ± 1.7	7.6 ± 1.6	0.12

AIS, ASIA Impairment Scale.

**Table 3:** Study-related adverse event.

	Placebo (n = 9)	Brivaracetam (n = 11)
Participants reporting study-related adverse events, n (%)	3 (33%)	8 (73%)
Serious adverse events (possibly related to study)		
Hospitalization due to nausea, vomiting, and altered mental status, n (%)	0 (0%)	1 (9%)
Nonserious adverse events (related or possibly related to study)		
Reported gastrointestinal symptoms, n (%)	**0 (0%)**	**5 (45%)**
Constipation, n (%)	0 (0%)	1 (9%)
Diarrhea, n (%)	0 (0%)	1 (9%)
Nausea/vomiting, n (%)	0 (0%)	4 (36%)
Stomach pain, n (%)	0 (0%)	3 (27%)
Reported neurological symptoms, n (%)	**3 (33%)**	**5 (45%)**
Dizziness, n (%)	0 (0%)	2 (18%)
Headache, n (%)	1 (11%)	1 (9%)
Brain fog/slowed speech, n (%)	0 (0%)	1 (9%)
Drowsiness, n (%)	0 (0%)	2 (18%)
Hand tremor, n (%)	1 (11%)	0 (0%)
Hand tremor, n (%)	1 (11%)	0 (0%)
Reported fatigue, n (%)	**2 (22%)**	**5 (45%)**
Reported psychological symptoms, n (%)	**2 (22%)**	**3 (27%)**
Depression/lack of motivation, n (%)	0 (0%)	1 (9%)
Anxiety, n (%)	0 (0%)	2 (18%)
Irritability, n (%)	1 (11%)	0 (0%)
Euphoria, n (%)	1 (11%)	0 (0%)
Reported vasomotor symptoms, n (%)	**1 (11%)**	**0 (0%)**
Hot flashes, n (%)	1 (11%)	0 (0%)
Reported arthralgias, n (%)	**0 (0%)**	**1 (9%)**

Three participants were withdrawn before study completion for unrelated medical issues (2 in the brivaracetam group who underwent surgery after starting the study: IDs 4049 and 4005, and 1 in the placebo arm with unrelated chest pain: ID 4044). One participant in the brivaracetam group was withdrawn before study completion due to hospitalization for nausea/vomiting that was potentially study related (ID 4043). One participant in the brivaracetam group stopped taking the medication and was withdrawn from the study without completing end-of-study testing or providing a pain diary (ID 3014). One participant in the brivaracetam group received the wrong drug formulation due to a pharmacy error (100 mg tablets split into 50 mg tablets: ID 3002) and was removed from the analysis. One participant in the placebo group (ID 4029) completed the study early due to no perceived benefit but completed 59 days of the pain diary. His end-of-study testing was completed early (day 59 instead of day 90), and he was included in the analysis. Two participants in the placebo group (IDs 3005 and 3009) and 1 in the brivaracetam group (ID 3015) reported 9–10/10 worst pain during prescreening but lower levels during baseline testing. They were included in the analysis. One participant in the brivaracetam group was not included in the repeated measures analysis of daily worst pain score from the pain diary due to insufficient data (ID 4037). In all, 14 participants (8 placebo and 6 brivaracetam) were included in the final analyses.

### 2.2. Primary and secondary outcomes

**Figure 2.**
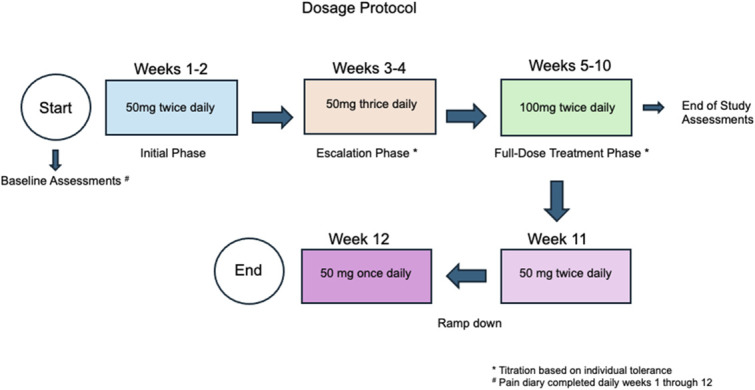
Timing of dosing and assessments.

Primary and secondary outcome measures were assessed for both groups at baseline and after the treatment period but before drug ramp down (see *Brivaracetam or Placebo Administration,* below). Primary outcome measures were pre-to postintervention (Fig. 2) change pain (worst, least, average, right now, composite) in the last 24 hours assessed by the Brief Pain Inventory,^7^ change in daily worst pain using the pain diary, and change in satisfaction with life using the Satisfaction with Life Scale (SWLS).^5^ Secondary outcomes included change in neuropathic pain with the International Spinal Cord Injury Pain Basic Dataset (V2.0),^22^ presence of neuropathic pain with the Douleur Neuropathique 4 Questions (DN4) tool,^6^ change in mood using the Patient Health Questionnaire (PHQ-9),^16^ change in sleep using the Pittsburgh Sleep Quality Index,^1^ change in self-efficacy using the Moorong Self-Efficacy Scale,^19^ change in kinesiophobia using the Tampa Scale for Kinesiophobia,^17^ change in pain catastrophizing using the Pain Catastrophizing Scale,^17^ and change in perceived disability using the Perceived Disability Index.^15^ Participants were asked about Intervention Expectations (at the start of study participation: What are your expectations with the study drug? At the end of study participation: What were your experiences with the study drug?) and Study Expectancies (How do you think your pain will change in 3 months, compared to your current level of pain?). The Satisfaction with Life Scale, Pittsburgh Sleep Quality Index, Tampa Scale for Kinesiophbia, Moorong Self-efficacy Scale, Pain Catastrophizing Scale, Perceived Disability Index, Penn Spasm Frequency Scale, Study Expectation, and Intervention Expectation outcomes were collected by self-reported REDCap survey. The other assessments were collected in person, over the phone, or by secure Zoom. Participants were instructed to shade neuropathic pain with red on the Brief Pain Inventory diagram. For those completing assessments virtually, the research assistant shaded the diagram with red for the participant with visual confirmation via Zoom. In addition, weekly updates were collected via self-reported REDCap survey or over the phone for reporting of hospitalization or illness that might affect dosing compliance, medication changes including change in pain medication use, or drug intolerance or side effects including changes in aggression or irritability, change in anxiety, hallucinations or delusions, or change in severity of depressive symptoms or suicidality (via PHQ-9).

### 2.3. Sample size calculations

No sample size calculations were conducted as this was a pilot clinical trial. A convenience sample of 24 participants was planned as this sample size would be sufficient to demonstrate a significant 0.6-point difference in worst pain between the 2 groups (80% power, 5% first-order risk). This size is also sufficient to adequately power a subsequent efficacy trial.

### 2.4. Medical clearance and secondary screening

Trained coordinators screened for eligibility before consent. After confirming eligibility, participants were required to obtain medical clearance to participate in this study from their medical provider. This included screening for liver dysfunction, renal insufficiency, and pregnancy, as brivaracetam treatment is not safe for those who are pregnant, have liver disease, or kidney dysfunction. Women who were menopausal or had not had a menstrual period for at least 12 months and women who had had a hysterectomy were not required to take pregnancy tests. Because brivaracetam treatment can increase risk of suicidality, we also screened for active suicidality. Final eligibility was determined by the study Principal Investigators. Participants deemed eligible and who had received medical clearance were then enrolled in the study.

### 2.5. Brivaracetam or placebo administration

**Figure 3.**
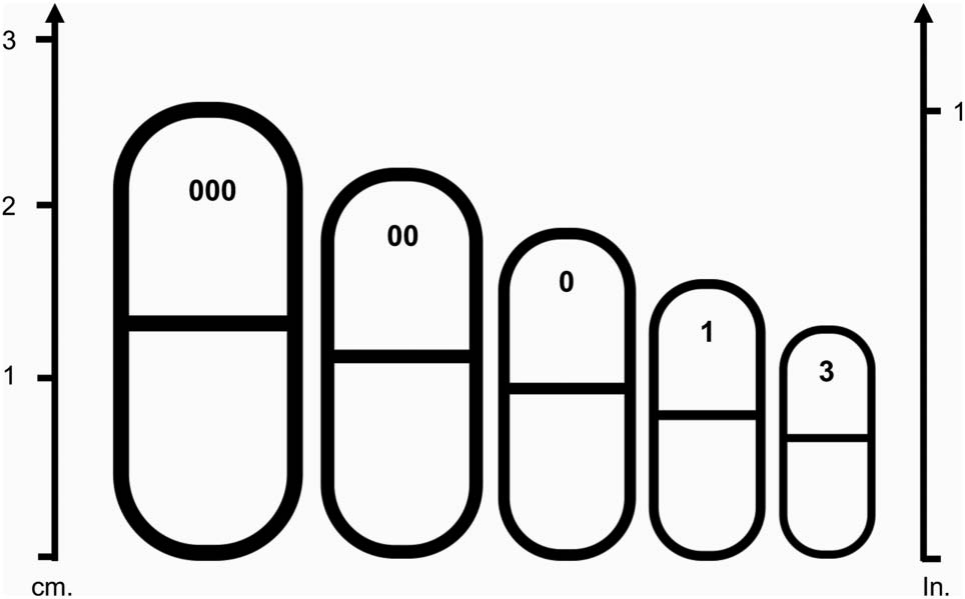
Capsule sizes. Brivaracetam and placebo drugs were compounded into 00 capsules (50 mg pills) and 000 capsules (100 mg pills). Sizes are shown in inches and centimeters.

Brivaracetam or placebo were compounded into a gelatin capsule by the study compounding pharmacy with identical appearance, consistency, and inactive ingredients (microcrystalline cellulose and silica gel). Participants and study staff could not distinguish brivaracetam from placebo. Drug dosage was individually titrated for each participant with a goal of 100 mg BID according to the dose escalation and ramp-down protocol (Fig. 2). The 50-mg tablets (brivaracetam and placebo) were compounded in a size 00 capsule, and the 100-mg tablets (brivaracetam and placebo) were compounded into a 000 capsule (Fig. 3). Participants were allowed to reduce the dose of brivaracetam if they experienced unacceptable drug side effects. In the case of drug intolerance, the dose was reduced to the last tolerated dose. For intolerance at 100 mg twice daily, the dose was reduced to 50 mg 3 times a day. For intolerance at 50 mg 3 times a day, the dose was reduced to 50 mg twice daily. A dose of 50 mg twice a day or higher was required to complete the trial. Once a stable dose was achieved, participants remained on this dose until the ramp down period at the end of the study. Because rapid withdrawal of anticonvulsant medications can lower the seizure threshold for those prone to seizures, study drug was gradually ramped down before discontinuation. The placebo dose was titrated and dispensed according to the same escalation and ramp down protocol as brivaracetam.

All participants received a medication and pain diary at the baseline visit. Because drug tolerance (including the ability to swallow the capsule) and study retention rates were unknown in individuals with SCI, and to minimize drug wasting and reduce drug costs, we dispensed medication via overnight mail in small installments. We overnight mailed each participant a 14-day supply during ramp up followed by a 10-day supply and then a 38-day supply once tolerance was confirmed. Similarly, ramp down doses were sent in weekly installments.

### 2.6. Variable definition

Clinical and demographic information was obtained by questionnaire at the time of enrollment and updated at the end of the study. Presence of neuropathic pain was determined by the International Spinal Cord Injury Pain Basic Dataset. Pain severity from the Brief Pain Inventory was considered as continuous variables (0–10). Pain interference domains were considered separately and as an interference composite without the walking domain. Daily pain ratings were considered as continuous variables (0–10). Neuropathic pain scores (International Spinal Cord Injury Pain Basic Dataset) were considered as continuous variables (0–10).

### 2.7. Statistical analysis

All analyses were performed using SAS 9.4 (SAS Institute, Inc., Cary, NC) or Stata, version 15 (StataCorp, College Station, TX). T-tests or χ^2^ were used to compare subject characteristics as appropriate and to assess postintervention changes in outcomes. Group differences in daily worst pain severity were assessed using repeat measures. Although many tests were conducted, no adjustments for multiple testing were done due to the small sample size. Differences were considered statistically significant for *P* < 0.05. The percentage of participants achieving 30% and 50% worst pain reduction for 1 or more study days was calculated from the 1,024 daily worst pain ratings (baseline worst pain score-daily worst pain score).

## 3. Results

### 3.1. Participant characteristics

Participant characteristics are presented in Table 2. All participants reported at least 1 and up to 3 at-level or below-level neuropathic pains at baseline testing. Participants in the intervention arm had higher “worst pain in the past 24 hours” and “pain right now” than the placebo group (*P* = 0.03). There were no other significant differences in demographics or injury characteristics between the 2 groups.

### 3.2. Study feasibility

Study retention was 70% with 14 of the 20 participants enrolled receiving at least 1 medication dose. Protocol adherence was high with 13 participants completing daily pain diaries, self-reported REDCap surveys, weekly check-ins, and primary/secondary outcome assessments. All participants were able to engage in Zoom study visits. Overnight medication delivery was effective with no instances of missing medication packages or failed delivery. Four participants disliked the compounded capsule size, but all were able to tolerate swallowing both pill sizes.

### 3.3. Adverse events

Serious and nonserious study-related adverse events are reported in Table 3. Side effects were more common in the brivaracetam group (73%, n = 8 vs 33%, n = 3) and were all expected based on the package insert/potential side effects. None developed active suicidal ideation while taking brivaracetam or placebo. One in the placebo group reported increased irritability (ID 3009) and 2 in the brivaracetam group reported increased anxiety (ID 4009 and ID 3034). One in the brivaracetam group (ID 4009) was unable to tolerate the maximum dose of 100 mg twice day due to fatigue and dizziness. After 2 days, this participant dropped down to 50 mg twice a day and completed the study at this dose. Two in the brivaracetam group (IDs 4043 and 4005) could not tolerate 100 mg twice daily due to nausea/diarrhea (4043: maximum tolerated dose 50 mg 3 times a day) or dizziness while being treated for a urinary tract infection (ID 4005: maximum tolerated dose 50 mg twice a day). There was 1 serious adverse event in the brivaracetam group (ID 4043: hospitalization due to nausea/vomiting) that was considered possibly study related. This participant was withdrawn at the time of hospitalization. For nonserious adverse events, 45% (n = 5) in the brivaracetam group reported GI symptoms (constipation, diarrhea, nausea/vomiting, and stomach pain). In most cases, GI symptoms improved or resolved with adjustments in the timing of the dosing (not taking at the same time as other medications, dosing before bed, or dosing with food). Eight reported more than 1 symptom on the same or different days (Table 4).

**Table 4:** Report of multiple side effects on the same or different days.

Subject ID	Side effects
Placebo	
3009	Headache, fatigue, irritability, and hot flashes
4033	Fatigue and euphoria
Brivaracetam	
3003	Nausea, constipation, and depressed mood
3014	Fatigue and headache
3031	Fatigue, drowsiness, and stomach pain
3034	Arthralgia, nausea with vomiting, and anxiety
4005	Fatigue, drowsiness, and dizziness
4009	Nausea, dizziness, fatigue, and slowed speech

### 3.4. Pain Reduction

**Figure 4.**
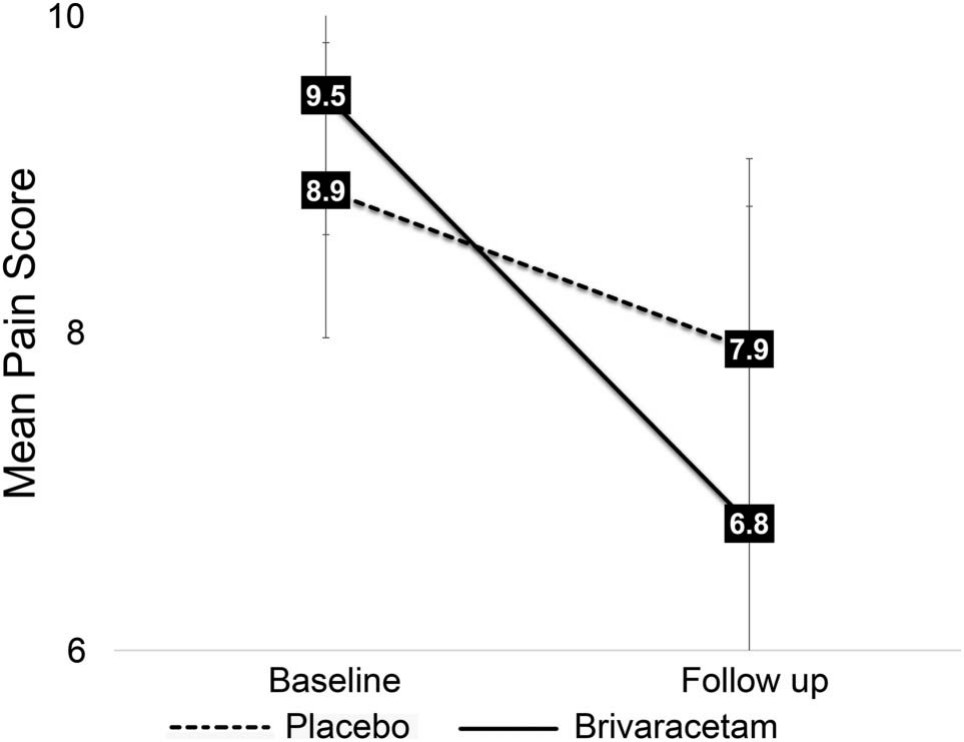
Reduced SCI-related neuropathic pain with brivaracetam treatment. Repeated measures analysis of worst daily pain score (1,024 data points) demonstrated significant differences between treatment groups (β = −1.7, 95% confidence interval: −1.9 to −1.4, *P* < 0.001). SCI, spinal cord injury.

**Figure 5.**
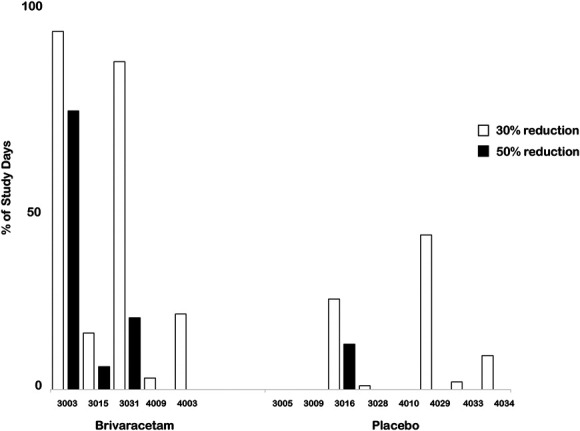
Percentage of treatment days with at least 30% or at least 50% pain reduction. Participants in the brivaracetam group experienced greater degrees of pain reduction for more treatment days than the placebo group.

When considering 1,024 data points from the daily pain diary, we observed a 2.7-point reduction in daily worst pain in the brivaracetam group compared to a 1-point reduction in the placebo group (β = −1.7, 95% confidence interval: −1.9 to −1.4, Fig. 4, *P* < 0.001). One hundred percent in the brivaracetam group achieved 30% reduction in pain on 1 or more days (mean of 40% of study days) compared to 60% in the placebo group (mean of 10% of study days, Fig. 5). Approximately 60% in the brivaracetam group achieved 50% pain reduction (mean of 20% of study days) compared to 12.5% in the placebo group (mean of 2% of study days, Fig. 5). There were no differences in least or average pain daily ratings (*P* = 0.1–0.4).

When considering postintervention change in pain as assessed by the Brief Pain Inventory (Table 5), participants in the brivaracetam group reported a mean 0.7 ± 1.6-point reduction in “pain right now” compared to a 1.0 ± 1.2-point increase in the placebo group (*P* = 0.04). Findings were similar but did not reach statistical significance when considering “worst pain” (−1.8 ± 1.5 vs −0.8 ± 1.2, *P* = 0.1), “least pain” (−0.2 ± 1.0 vs 0.5 ± 1.2, *P* = 0.3), “average pain” (−0.5 ± 1.9 vs −0.3 ± 0.7, *P* = 0.8), and composite pain (−0.8 ± 1.1 vs 0.1 ± 0.5, *P* = 0.1). Participants in the brivaracetam group reported a mean 3.7 ± 3.1-point reduction in pain interference with mood compared to the 0.6 ± 1.7-point reduction in the placebo group (*P* = 0.04). Findings were similar but did not reach statistical significance when considering the other pain interference domains, neuropathic pain severity by the DN4, composite interference score, pain catastrophizing, and depressive symptoms (Table 5, *P* = 0.07–0.8). There was no difference in postintervention change in satisfaction with life between groups (Table 5, −0.4 ± 4.5 vs −0.4 ± 5.7, *P* = 0.8).

**Table 5:** Mean postintervention changes for primary and secondary outcomes.

	Placebo (n = 8)			Brivaracetam (n = 6)			*P*
	Pre	Post	Difference	Pre	Post	Difference	
Primary outcomes							
Pain (brief pain inventory)							
Worst pain	8.9 ± 1.0	8.1 ± 1.6	−0.8 ± 1.2	9.7 ± 0.8	7.8 ± 1.7	−1.8 ± 1.5	0.1
Least pain	5.1 ± 3.3	5.6 ± 2.4	0.5 ± 1.2	4.8 ± 3.2	4.7 ± 3.4	−0.1 ± 1.0	0.3
Average pain	6.7 ± 1.8	6.4 ± 1.9	−0.3 ± 0.7	7.5 ± 1.6	7.0 ± 1.8	−0.5 ± 1.9	0.8
Pain right now	5.4 ± 2.3	6.4 ± 2.7	1.0 ± 1.2	7.8 ± 2.7	7.0 ± 2.5	−0.8 ± 1.6	**0.04**
Composite pain	6.4 ± 1.7	6.5 ± 1.9	0.1 ± 0.5	7.3 ± 1.7	6.5 ± 2.3	−0.8 ± 1.1	0.1
Satisfaction with life	17.9 ± 7.0	18.3 ± 8.4	−0.4 ± 4.5	20.2 ± 5.6	19.8 ± 8.1	−0.4 ± 5.7	0.8
Secondary outcomes							
Pain interference (BPI)							
General activity	5.9 ± 3.6	6.4 ± 3.0	0.5 ± 2.4	5.5 ± 4.1	4.8 ± 4.1	−0.7 ± 5.5	0.6
Mood	5.8 ± 2.8	5.1 ± 3.6	−0.6 ± 1.7	7.0 ± 3.8	3.3 ± 2.3	−3.7 ± 3.1	**0.04**
Normal work	5.3 ± 4.2	5.9 ± 3.9	0.6 ± 3.4	6.2 ± 3.5	5.7 ± 3.9	−0.5 ± 1.6	0.5
Relations with others	4.4 ± 4.4	4.9 ± 3.5	0.5 ± 3.4	6.2 ± 3.3	3.3 ± 3.8	−2.8 ± 2.9	0.07
Sleep	5.8 ± 3.5	4.3 ± 3.8	−1.5 ± 2.1	7.0 ± 2.4	6.2 ± 3.7	−0.8 ± 1.8	0.5
Enjoyment of life	6.6 ± 3.5	6.4 ± 3.4	−0.2 ± 3.8	5.2 ± 4.3	4.3 ± 3.7	−0.8 ± 0.6	0.7
Composite (minus walking)	5.6 ± 3.2	5.5 ± 3.2	−0.1 ± 2.1	6.9 ± 1.3	5.4 ± 2.4	−1.5 ± 1.7	0.2
Pain (ISCI)							
Worst neuropathic pain	7.0 ± 1.9	6.6 ± 1.9	−0.4 ± 1.1	8.7 ± 0.8	8.2 ± 1.7	−0.5 ± 2.2	0.9
Composite neuropathic pain	6.9 ± 1.9	6.5 ± 1.9	−0.4 ± 1.1	8.7 ± 0.7	8.0 ± 1.8	−0.7 ± 2.2	0.1
All pain sites	11.6 ± 11.0	10.9 ± 11.1	−0.8 ± 5.8	22.2 ± 14.1	17.2 ± 10.3	−5.0 ± 6.3	0.2
Pain interference (ISCI)							
Day-to-day activities	7.5 ± 3.2	7.1 ± 2.7	−0.4 ± 2.0	7.3 ± 3.5	6.7 ± 3.4	−0.6 ± 1.4	0.8
Mood	7.3 ± 2.3	6.0 ± 3.1	−1.3 ± 1.8	7.3 ± 2.3	5.2 ± 3.4	−2.2 ± 2.2	0.4
Sleep	5.8 ± 4.0	4.8 ± 3.2	−1.0 ± 2.3	7.3 ± 2.9	6.7 ± 3.7	−0.6 ± 0.8	0.7
Composite	6.8 ± 2.5	6.0 ± 2.4	−0.9 ± 1.7	7.3 ± 2.2	6.2 ± 3.0	−1.1 ± 1.4	0.7
Neuropathic pain symptoms (DN4)	6.3 ± 1.7	6.4 ± 1.7	0.1 ± 0.6	7.7 ± 2.1	6.8 ± 2.8	−0.8 ± 2.0	0.3
Pain catastrophizing scale							
Rumination subscale	10.3 ± 4.2	8.8 ± 4.2	−1.5 ± 2.4	10.3 ± 6.0	9.5 ± 5.5	−0.8 ± 3.2	0.7
Magnification subscale	3.0 ± 2.5	3.6 ± 2.9	0.6 ± 3.0	4.7 ± 4.0	2.8 ± 3.1	−1.8 ± 3.8	0.2
Helplessness subscale	10.6 ± 6.7	10.4 ± 6.9	−0.2 ± 4.5	12.5 ± 6.9	9.2 ± 6.0	−3.3 ± 3.3	0.2
Total score	23.9 ± 12.1	22.8 ± 13.5	−1.1 ± 8.7	27.5 ± 16.4	21.5 ± 14.1	−6.0 ± 9.0	0.3
Depressive symptoms (PHQ-9)	9.5 ± 5.6	9.9 ± 9.7	0.4 ± 7.5	8.0 ± 5.1	8.0 ± 5.1	−1.2 ± 5.2	0.7
Kinesiophobia (TKS)							
Avoidance	16.3 ± 1.4	15.5 ± 2.1	−0.8 ± 2.9	16.3 ± 3.3	15.7 ± 3.8	−0.7 ± 3.3	0.9
Somatic focus	13.3 ± 2.5	12.9 ± 1.6	−0.4 ± 3.4	13.8 ± 2.6	13.5 ± 2.1	−0.3 ± 4.0	1.0
Total score	39.0 ± 9.2	39.8 ± 5.5	0.8 ± 5.0	42.2 ± 7.3	41.3 ± 7.6	−0.8 ± 7.4	0.6
Sleep quality (PSQI)	10.9 ± 3.9	11.1 ± 4.8	−0.3 ± 3.2	13.2 ± 4.5	11.7 ± 4.2	−1.7 ± 2.5	0.4
Self-efficacy (Moorong)	82.4 ± 18.2	78.6 ± 18.9	−3.8 ± 10.8	103.2 ± 5.8	100.2 ± 11.0	−3.0 ± 8.8	0.9
Perceived disability index	44.3 ± 14.3	44.4 ± 14.8	0.1 ± 14.0	39.5 ± 10.1	34.3 ± 19.0	−5.2 ± 12.0	0.5

Bold values indicate *P* ≤ 0.05

DN4, Douleur Neuropathique 4; PHQ-9, Patient Health Questionnaire-9; BPI, Brief Pain Inventory; ISCI: International Spinal Cord Injury Pain Basic Dataset (V2.0); PSQI: Pittsburgh Sleep Quality Index; TKS: Tampa Scale for Kinesiophobia.

**Figure 6.**
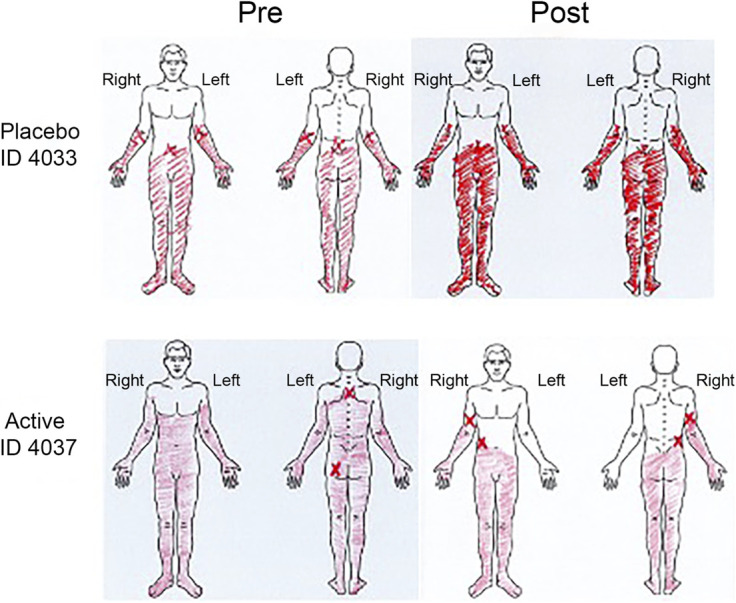
Reduced distribution of SCI-related neuropathic pain with brivaracetam treatment. Participants in the brivaracetam group experienced greater reduction in pain distribution than the placebo group. SCI, spinal cord injury.

Four participants in the brivaracetam group reported reduced pain distribution by the Brief Pain Inventory Pain Diagram (Fig. 6 ID 4049 and Supplemental Figure 1a, http://links.lww.com/PR9/A321 IDs 3031, 4009, 4037) compared to 1 in the placebo group (Supplemental Figure 1b, http://links.lww.com/PR9/A321 ID 4034). The brivaracetam group also reported a greater reduction in the number of all pain locations (−5.0 ± 6.3 vs −0.8 ± 5.8, *P* = 0.2) and neuropathic pain only pain locations (−5.3 ± 6.4 vs 0.3 ± 6.6, *P* = 0.1) assessed by the International SCI Pain Basic Dataset compared to the placebo group.

Regarding postintervention expectations (Table 6), 2 in the placebo group believed that they had received brivaracetam, whereas 5 in this group reported no pain relief from the drug or that they believed they were in the placebo group. In contrast, 1 in the brivaracetam group reported no pain relief and 4 participants in this group reported neuropathic pain relief to varying degrees.

**Table 6:** End of study intervention expectations.

Subject ID	What were your experiences with the study drug?
Placebo	
3005	It was helpful for me, and I hope it's helpful for others. I've learned more about myself. (Participant received prescription from PCP after study ended)
3009	I was open-minded but I don't think it [study drug] did anything. Same boat and that boat sinks every once in a while
3016	I didn't notice a difference with being on the study drug
3028	It's been easy. A piece of cake
4010	I feel that I did have the drug
4029	My engagement with the drug was not what I hoped for. I do not feel like I received pain relief and felt that it had a negative cognitive impact and caused a lack of mental clarity
4033	The medication seemed to make me more tired than I already am. No relief in pain
4034	At the end of the study, I had somewhat concluded I was most likely on the placebo or that I had zero side effects and zero benefit feeling as well
Brivaracetam	
3003	It worked! (participant received prescription from PCP after study ended)
3015	The throbbing/pulsating pain remained the same. I have not experienced the electrifying/shocking flutter pain since before starting the study, I did not experience side effects from the drug. (Participant requested prescription from PCP after study ended)
3031	NA
3034	I was hopeful that I would get pain relief, but nothing changed
4009	At the beginning, there were some side effects that got better after figuring out a good dose. In the middle when I tried taking a higher dose and experienced little to no appetite and 1 time that I experienced high anxiety and running thoughts. It was a lot to get used to with being more tired and taking breaks and short naps to get through the day. I didn't have much for neuropathy pain relief. I would say it wasn't as bad in my upper legs as it had been previous. My lower leg pain remained the same for the most part, maybe slight decrease but not significant
4037	I feel that 50 mg 3 times a day works best for me. (Participant received prescription from PCP after study ended)

PCP, Primary Care Physician.

### 3.5. Change in depressive symptoms

No participant reported passive suicidal ideation at study baseline. Participants in the brivaracetam group experienced a reduction in depressive symptoms, whereas participants in the placebo group experienced an increase in depressive symptoms (−1.2 ± 5.2 vs 0.4 ± 7.5, *P* = 0.7). Two in the placebo group (IDs 3028 and 4033) and 1 in the brivaracetam group (ID 3003) reported moderately severe depressive symptoms at baseline, and these symptoms improved to mildly depressive symptoms in 2 participants (IDs 3003 brivaracetam and 3028 placebo) and worsened to severe in 1 participant (ID 4033 placebo) at postintervention testing. One participant in the placebo group (ID 4029) worsened from mild depressive symptoms to severe.

### 3.6. Change in pain medication use

Concurrent pain medication use was frequent in both groups (Table 7) with gabapentinoids (79%), opioids (64%), and cannabinoids (50%) being the most common. Changes in medication use are presented in Table 8. Increased dosing of narcotics or the addition of ketamine were more common in the placebo group. Two participants in the placebo group (IDs 3016 and 4034) and 2 in the brivaracetam group (IDs 3015 and 4027) reported no change in pain medication use and are not included in Table 8.

**Table 7:** Preintervention pain medication use.

ID	Medications					
	Narcotics or ketamine	Analgesics (nonnarcotic)	Neuropathic pain meds	Spasmolytics	Antidepressants	CBD/THC
Placebo						
3005	Ketamine	Acetaminophen	Gabapentin	Baclofen	Amitriptyline Duloxetine	CBD/THC gummies
3009	Oxycodone/acetaminophen	Acetaminophen Ibuprofen Tramadol	Gabapentin	Diazepam		
3016	Oxycodone/acetaminophen	Acetylsalicylic acid Ibuprofen	Gabapentin	Baclofen		
3028			Gabapentin Pregabalin	diazepam Lorazepam	Mirtazapine	CBD/THC gummies
4010	Oxycodone/acetaminophen	Acetylsalicylic acid Ibuprofen Acetaminophen Naproxen		Baclofen Diazepam		Medical cannabis
4029	Oxycodone	Acetaminophen	Gabapentin Pregabalin		Bupropion	Medical cannabis
4033	Ketamine	Acetaminophen	Gabapentin		Duloxetine	Medical cannabis
4034	Oxycodone/acetaminophen		Gabapentin	Baclofen		Medical cannabis
Brivaracetam						
3003	Methadone	Acetaminophen Ibuprofen		Baclofen	Duloxetine	Marijuana
3015	Oxycodone fentanyl patch	Ibuprofen		Alprazolam Zolpidem Botulinum toxin		
3031		Acetaminophen Ibuprofen	Gabapentin	Baclofen Clonazepam		
3034	Methadone	Acetylsalicylic acid Acetaminophen Ibuprofen	Gabapentin			
4009		Acetaminophen Ibuprofen Naproxen	Gabapentin	Baclofen		
4037	Hydrocodone		Gabapentin	Baclofen	Amitriptyline	

CBD, cannabidiol; THC, tetrahydrocannabinol.

**Table 8:** Postintervention reported change in medication use.

ID	Medications					
	Narcotics or ketamine	Analgesics (nonnarcotic)	Neuropathic pain meds	Spasmolytics	Antidepressants	CBD/THC
Placebo						
3005			Reduced gabapentin		Increased amitriptyline, reduced duloxetine	
3009	Reduced oxycodone/acetaminophen					
3028	Started ketamine infusions				Stopped mirtazapine	
4010	Increased oxycodone/acetaminophen	Stopped acetylsalicylic acid		Increased baclofen		
4029	Stopped oxycodone, started oxycodone/acetaminophen		Increased pregabalin		Stopped bupropion	
4033	Increased ketamine		Increased gabapentin			
Brivaracetam						
3003		Stopped acetaminophen and ibuprofen				Stopped marijuana
3031		Increased acetaminophen dose				
3034	Added oxycodone/acetaminophen	Added meloxicam	Reduced gabapentin, added pregabalin		Added nortriptyline	
4009		Stopped naproxen, added diclofenac				

CBD, cannabidiol; THC, tetrahydrocannabinol.

## 4. Discussion

We conducted a multicenter, double-blinded, placebo-controlled pilot clinical trial testing brivaracetam to reduce SCI-related neuropathic pain. We found that our study design, including national recruitment, was feasible and effective. Study retention and protocol adherence were both high. The drug was safe with no serious or unexpected adverse events. Although efficacy was not the focus of this pilot clinical trial, we did find that pain reduction was greater in the brivaracetam group compared to the placebo group. Importantly, some participants have requested brivaracetam prescriptions from their primary care physicians after the study ended and have successfully obtained insurance coverage to take this medication to treat neuropathic pain. We assessed pain outcomes using several tools to determine which tool would best capture potential changes in neuropathic pain severity and/or distribution. Although the findings were similar and complimentary across the various pain tools, we found the greatest differences in pain reduction using the pain diary. We also found significant differences in this small sample using the Brief Pain Inventory questions “pain right now” and pain interference with mood and consider these outcomes may have utility as primary outcome measures in future clinical trials. There is a lack of consensus regarding clinically meaningful changes in pain based on the 2008 IMMPACT Recommendations^7^ and the revised 2020 IMMPACT Recommendations,^21^ but 1-point, 2-point, 10%, and 20% thresholds have been proposed. It should be noted that neither statement is specific to SCI-neuropathic pain. Despite this lack of clarity in the field, our pilot daily pain diary data of ratings of worst pain exceed all thresholds for clinically meaningful reductions in pain put forth by the IMMPACT Recommendations. We, therefore, consider our pilot findings promising and supportive of future clinical trials using daily pain scores as a primary outcome.

We monitored mood, irritability, and anxiety by weekly self-report and a weekly follow-up phone or Zoom check-ins. None developed active suicidal ideation. Depressed mood is common in individuals with SCI.^10,23^ Plans to adequately address new active suicidal ideation is essential allowing clinical trials to proceed without excessively stringent exclusion criteria. Excluding participants with a history of passive suicidal ideation or a history of depressed mood would render a clinical trial such as this difficult to conduct, if not futile. In this study, participants in the brivaracetam arm experienced reduced depressive symptoms, and those in the placebo group experienced worsening depressive symptoms. Although this difference did not achieve statistical significance, the impact of brivaracetam on mood should be assessed in clinical trials powered to detect these changes.

We were able to calculate the number of days each participant experienced pain reduction of various magnitudes. Although it has been reported that the placebo effect creates challenges in clinical trials,^7,21^ in this study, more participants in the brivaracetam group reported achieving at least 30%, and 50% pain reduction for a greater percentage of study days compared to placebo. We queried postintervention expectations to assess for placebo effect. Our findings suggest that brivaracetam efficacy exceeds the placebo effect in SCI-neuropathic pain and that this can be adequately tested in fully powered clinical trials. The pain diary was advantageous in this study as it provided repeated daily assessments that spanned the entire duration of the study, including the ramp-up and ramp-down periods. In this study, we analyzed 1,024 daily worst pain ratings. This allowed us to detect reductions in daily pain score within days of the first dose in some participants. Based on this finding, a 1-month intervention after the ramp-up period might be sufficient to demonstrate efficacy, especially if participants provide daily pain diaries. This needs to be tested in future trials. Pain reductions were reported on the ramp-up dose. A lower dose may be as effective as the higher dose with a more favorable side-effect profile and at a significantly lower treatment cost. Although this pilot study was not designed to detect differences based on daily dose, we will use the findings to power a larger, dose–response clinical trial.

There are few clinical trials demonstrating efficacy to treat SCI-related neuropathic pain. Although levetiracetam failed to reduce neuropathic pain in a small study of adults with SCI,^9^ brivaracetam has 30 times the SV2A-binding affinity compared to levetiracetam.^12^ We hypothesize that our promising preliminary findings are due to 2 factors: (1) the increased SV2A-binding affinity and (2) our utilization of the daily pain diary. Fully powered, comparative efficacy clinical trials are needed to definitively determine if brivaracetam is more effective at reducing SCI-neuropathic pain than levetiracetam.

Individuals living with SCI may have multiple secondary health complications, including frequent illness and hospitalizations, poor sleep quality,^4^ and depressed mood.^10,23^ The factors contributing to the development and maintenance of neuropathic pain are poorly understood,^2^ but medical comorbidities and psychosocial factors may exacerbate the subjective experience of pain. Therefore, a daily pain diary may be a superior primary outcome to assess efficacy of pain treatments because it allows for statistical consideration of multiple data points over the course of the intervention period. Assessing pain at only 2 time points may diminish the ability to determine intervention efficacy as the outcome assessment may be missed due to unanticipated hospitalization or may be affected by an emerging acute illness or unrelated psychosocial factor such as poor sleep quality the preceding night.

There are limitations of the current study. Side effects are more common in the brivaracetam group, and this may unblind study staff and participants in future trials. This can be mitigated by not considering study arm when reviewing adverse events unless unblinding is deemed medically necessary. Three participants reported less severe pain at baseline testing than the minimum 9/10. Also, our protocol did not allow for assessment of pain scores before or after the intervention. Future studies may address these points. Despite these limitations, we conclude brivaracetam is well tolerated in adults with SCI-related neuropathic pain. Fully powered clinical trials are feasible and necessary to determine the magnitude of pain reduction expected with brivaracetam treatment and to optimize dosing.

## Disclosures

The authors have no conflict of interest to declare.

## Appendix A. Supplemental digital content

Supplemental digital content associated with this article can be found online at http://links.lww.com/PR9/A321.

## Supplementary Material

SUPPLEMENTARY MATERIAL
