# Supplementary figures and images for: Brivaracetam for spinal cord injury–related neuropathic pain: results of a pilot double-blinded, randomized, placebo-controlled clinical trial
Source: Pain Rep. 2025 Jun 16;10(4):e1301. doi: 10.1097/PR9.0000000000001301 (PMC12173286; doi:10.1097/PR9.0000000000001301)

Supplemental Figure 1a.

Pre

Post

3003

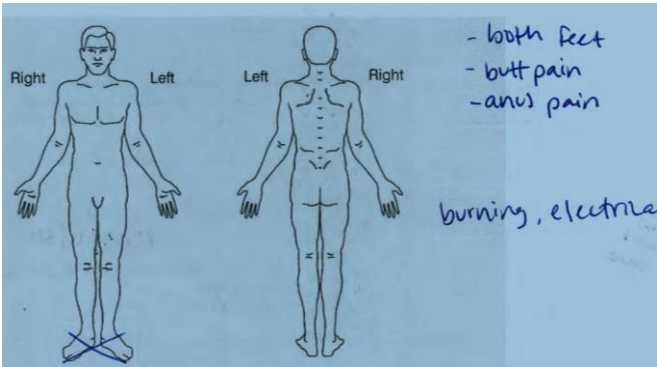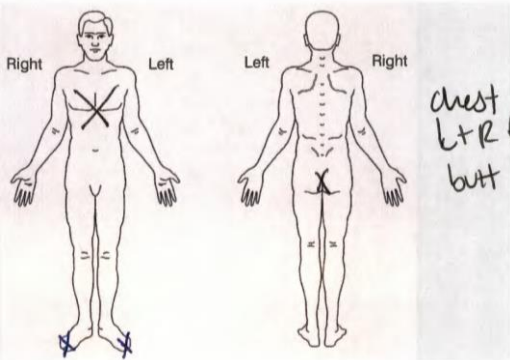

3031

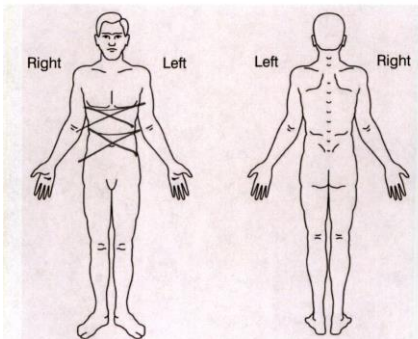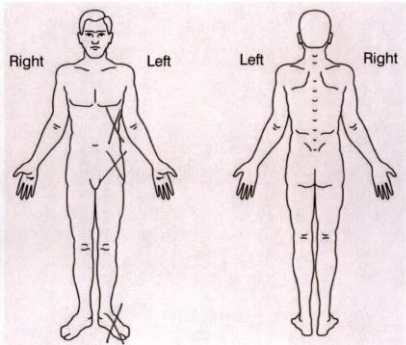

4009

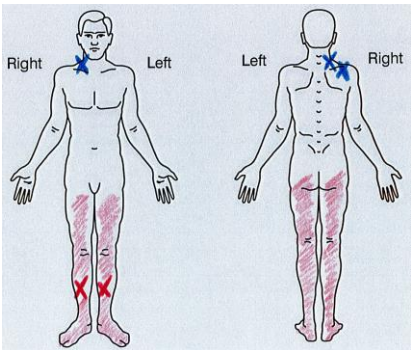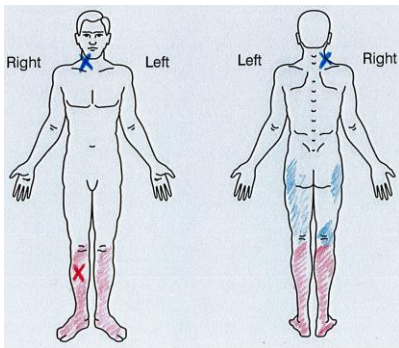

4037

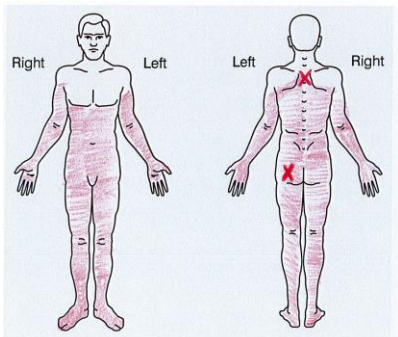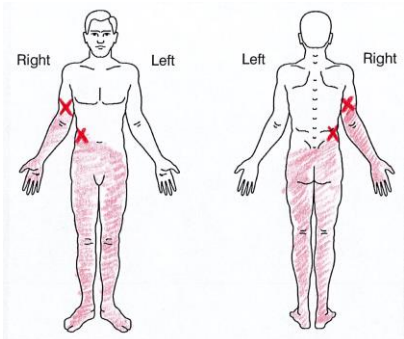

Supplemental Figure 1b.

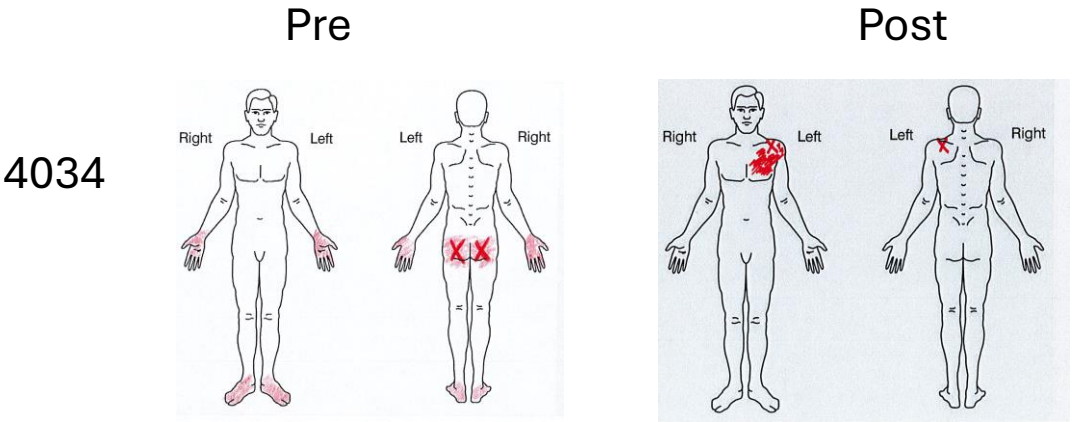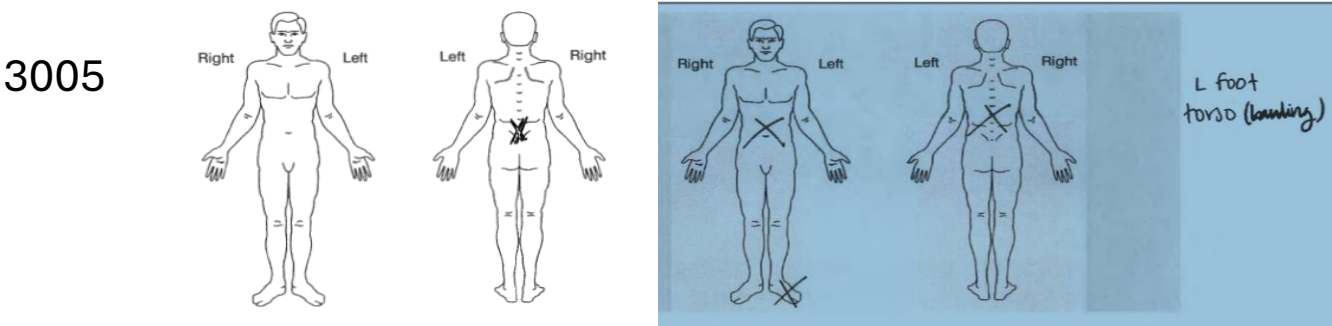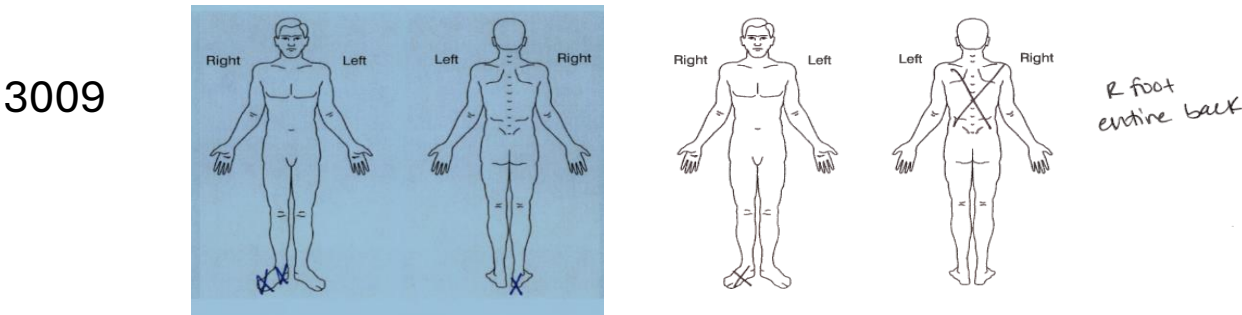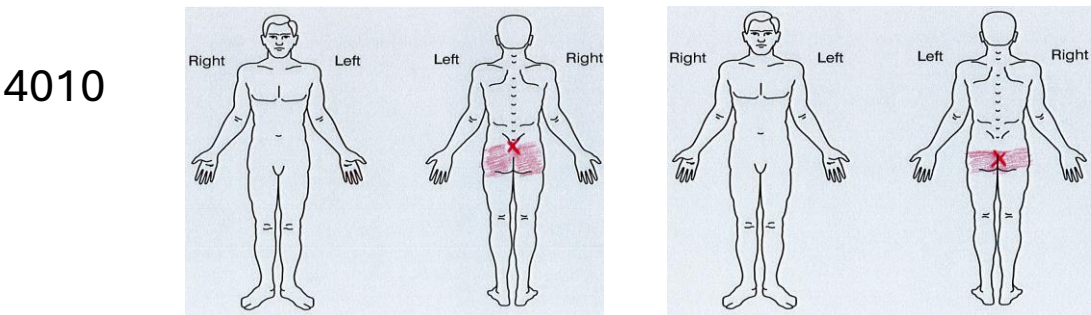

Pre

Post

4029

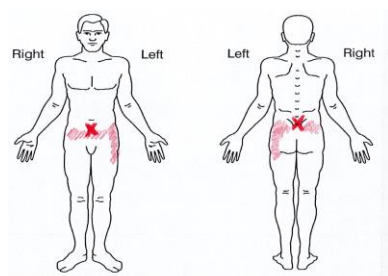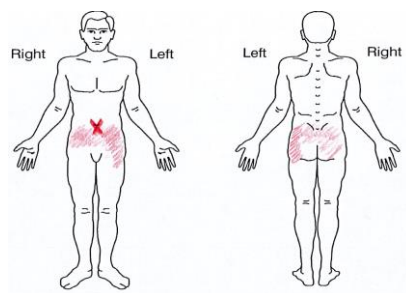

4033

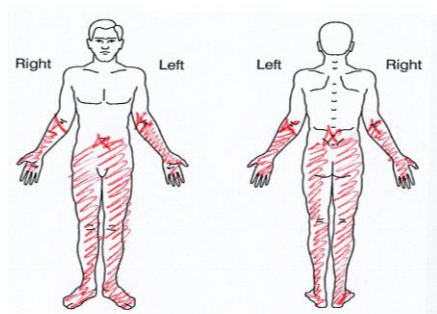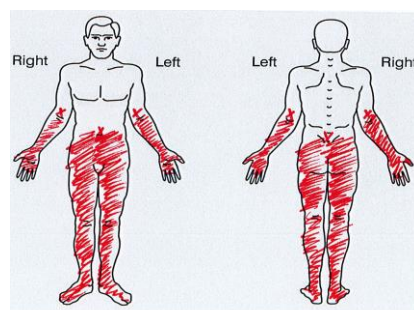

Supplement: SUPPLEMENTARY MATERIAL [file painreports-10-e1301-s001.pdf]
